# Occurrence of ^222^Rn and ^226,228^Ra in underground water and ^222^Rn in soil and their mutual correlations for underground water supplies in southern Greater Poland

**DOI:** 10.1007/s10653-020-00792-z

**Published:** 2021-01-28

**Authors:** Henryk Bem, Magdalena Długosz-Lisiecka, Daria Mazurek-Rudnicka, Piotr Szajerski

**Affiliations:** 1 Calisia University - Kalisz, Poland, Nowy Swiat 4, 62-800 Kalisz, Poland; 2 Institute of Applied Radiation Chemistry, Lodz University of Technology, Wroblewskiego 15, 93-590 Lodz, Poland

**Keywords:** ^222^Rn, ^226,228^Ra nuclides, Underground water, Drinking water, Liquid scintillation, Radon in soil

## Abstract

European Union Council Directive 2013/51/EURATOM recently sets out so-called indicator parameters for: radon, tritium and indicative dose of water intended for human consumption. The aim of this research was to elaborate an effective procedure for determination of radon and radium ^226,228^Ra isotopes (which are potentially the main contributors to the internal dose from drinking and cooking water) and to find the possible relationships between these radionuclides in underground water reservoirs and ^222^Rn concentration in the soil gas in their vicinity. The research was performed by applying a non-volatile and water-immiscible scintillation cocktail based on a pure diisopropylnaphthalene (Ultima Gold F: UGF), which allow for efficient radon extraction from 0.5 dm^3^ of water samples to 20 cm^3^ of scintillation phase and its direct determination with a detection limit of 5 × 10^–3^ Bq dm^−3^. The further preliminary concentration of 3 dm^3^ of crude water samples by evaporation to 0.5 dm^3^ samples led to the removal of all unsupported ^222^Rn activity and allowed the ^226^Ra determination via equivalent ^222^Rn detection after one-month samples storage using a low-background Triathler liquid scintillation counter in the α/β separation counting mode. Together with determination of ^226^Ra isotope in water samples, the simultaneous measurements of ^228^Ra and ^222^Rn radionuclides concentrations in water as well as ^222^Rn activity in the soil gas around the water supply sites were performed. The achieved limit of ^226^Ra detection was at a very low level of 10^–3^ Bq dm^−3^. The measured values of ^226^Ra concentration in 50 public underground water supply units for the Kalisz district of Poland were relatively low and ranged from below detection limit to 28.5 × 10^–3^ Bq dm^−3^ with arithmetic mean and median values of 12.9 and 12.2 × 10^–3^ Bq dm^−3^, respectively. Weak correlations were observed between activity concentrations of ^226^Ra and ^222^Rn in the crude water samples (*R*^*2*^ = 0.31) and ^222^Rn in water and its concentration in the nearby soil gas (*R*^*2*^ = 0.48).

## Introduction

Underground waters are important sources of drinking water worldwide. The necessity of implementing EU Council Directive 2013/51/EURATOM led to increased demand for measurements of radon, as well as ^226,228^Ra nuclides, in drinking water as potentially the main contributors to the total internal dose for the general public with regard to radioactive substances in water intended for human consumption (Gowing et al. 2019; Vasile et al. 2016). According to these recommendations, the so-called indicative reference value should not exceed 0.1 mSv per year (European Commission 2013). Radon, as it is relatively soluble, enters the water not from poorly dissolved radium salts but mainly from the decay of radium in soil and adjacent rocks (Porcelli and Swarzenski 2003; Wanty and Schoen 1992). Therefore, its (^222^Rn) world average concentrations in underground waters are generally much higher (29.1 Bq dm^−3^) than its mother nuclide, ^226^Ra, for which the observed average concentration was estimated at 87.2 mBq dm^−3^ (Florică et al. 2020; Girault et al. 2018; Loomis et al. 1988; Nazir et al. 2020).

Radon and its decay products are known to present a risk of lung cancer when they are inhaled (World Health Organization 2009, 2017). Although most of the radon, that enters indoor air, comes directly from soil (UNSCEAR 1982), however the domestic usage of water can be also a substantial source of this radionuclide in the indoor air in some circumstances (Harley et al. 2014). Generally, the radon risk caused by ingestion of drinking water is much lower than that from inhalation, and based on a daily water intake by a standard man and dose conversion factor, the EU Council recommended a relatively high parametric value of ^222^Rn activity equal to 100 Bq dm^−3^. However, more than 50% of groundwater samples from the Karkonosze granite area in Poland contain over 100 Bq dm^−3^ of ^222^Rn, and due to the 100 Bq dm^−3^ limit, they cannot be directly distributed for consumption through the public water supply networks (Przylibski et al. 2020).

Currently, of the several procedures described in the literature for direct indoor radon and radon in water measurements, liquid scintillation (LSC) still plays a leading role (Jobbágy et al. 2017). This technique, which utilizes the high solubility of the gaseous Rn in aromatic solvents (common scintillation solvents), was introduced by Horrocks five decades ago (Horrocks and Studier 1964), and different versions are still published (Bem et al. 1994; Cantaloub 2000; Cassette et al. 2006; Gowing et al. 2019; Idoeta et al. 2018; Nikolov et al. 2018; Prichard and Gesell 1977). In recent decades, a new class of liquid scintillation cocktails based on diisopropylnaphthalene with very low vapor pressure and excellent detection efficiency appeared on the market. In conjunction with a new generation of portable liquid scintillation counters, such a combination allows simple radon extraction from 10 cm^3^ water samples into 10 cm^3^ of water-immiscible scintillation cocktail, directly in 20 cm^3^ volume vials and two-phase counting, according to the Prichard and Gesell procedure (Prichard and Gesell 1977). The radon partition coefficients between the water phase and typical scintillation solvents were reported by Cantaloub, and the value for the UGF cocktail/water system is reasonably high, equal to 32 in ambient temperature (Cantaloub 2000).

The method of direct radon extraction to the scintillation cocktail has been successfully used to determine ^222^Rn in public underground drinking water supplies of the southern Greater Poland region (Bem et al. 2014). However, a calculated detection limit of that method equals 0.11 Bq dm^−3^, which was not sufficient for consecutive ^226^Ra determination in underground waters. These studies aimed to extend this method for ^226^Ra determination in drinking water after a sixfold preconcentration of the samples by its evaporation with the simultaneous removal of the initially present ^222^Rn nuclide and measure of ^222^Rn activity in equilibrium with ^226^Ra after one-month sample storage.

The results obtained using this method have been compared with the classical radium nuclide preconcentration procedure based on a coprecipitation of radium with barium and lead sulfates and γ-spectrometric determinations of ^226^Ra and ^228^Ra (via its decay product ^228^Ac). It also seems to be interesting to look for possible correlations between ^226^Ra or ^222^Rn concentration in crude (not pretreated) water and ^222^Rn concentration in the soil gas in the vicinity of water pumping stations (Cucoş et al. 2017; Moreno et al. 2018). The obtained data on ^226,228^Ra and ^222^Rn concentrations were also correlated with the physical parameters of the water samples and with concentrations of major and trace elements, to verify existence of the possible relationships. Since the majority of the examined water supply stations are situated near the main river in this region, the Prosna, determining the exact radium isotope ratios can be useful for scientific information concerning mechanisms and rates of water/rock interactions and the contribution of river water transport to groundwater (Sturchio et al. 2001). Such information can lead to establish better strategies for the use and quality protection of underground water reservoirs.

## Materials and methods

### *Water sampling*

**Fig. 1:**
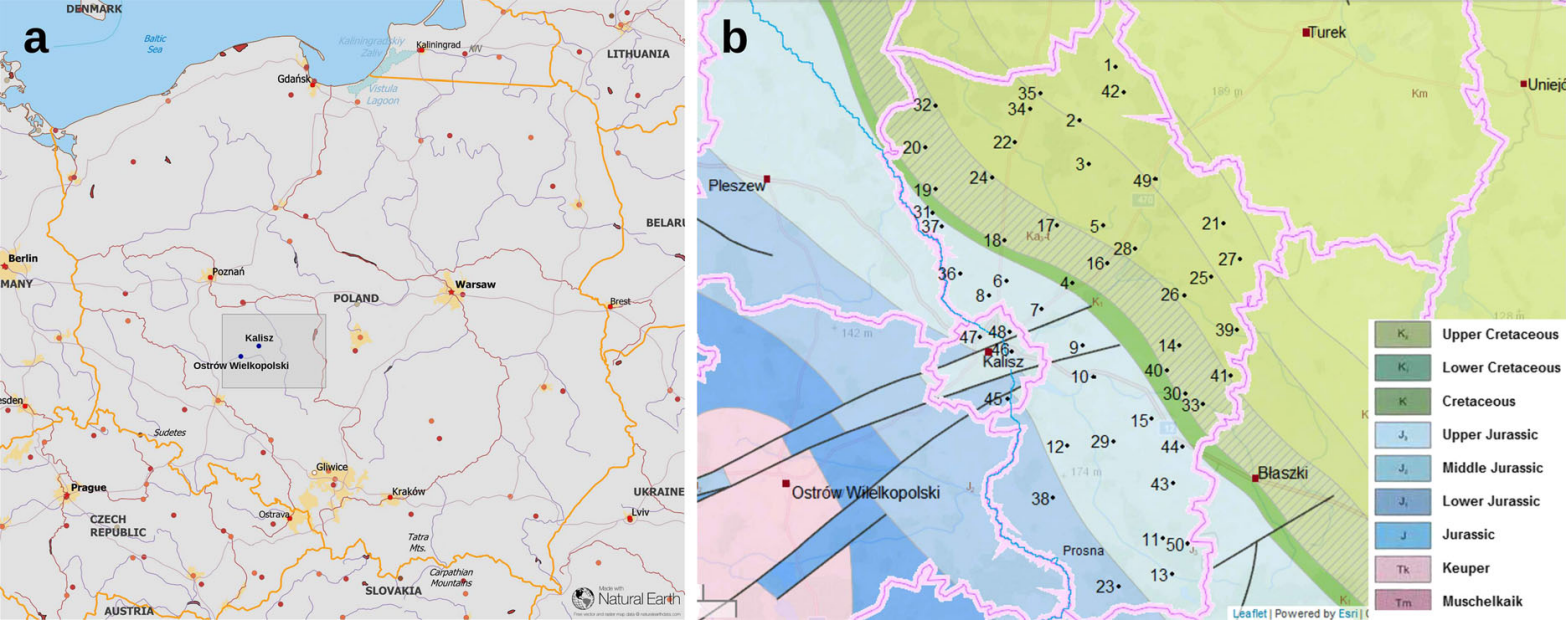
Kalisz district on the map of Poland (**a** made with Natural Earth, www.naturalearthdata.com) and location of the water sampling points (1–50) on the stratigraphic map of Kalisz region (**b** geolog.pgi.gov.pl)

The underground water samples were collected from 50 public groundwater supply units and local water distribution network sites situated in the Kalisz region of Greater Poland. Location of the sampling points is presented on the map of Poland and Kalisz district in Fig. 1. The samples were collected from appropriate water networks, before treatment and after the treatment procedure in the water treatment plants (WTP), after removing out about 10 L of water followed by a slow laminar flow into three 1.5-dm^3^ plastic bottles contained 0.1 ml concentrated nitric acid (to avoid radium adsorption). The samples were transferred to the laboratory with a delay not exceeding 1–2 days.

### ^*222*^*Rn and *^*226*^*Ra determination by extraction from 0.5 dm*^*3*^* flasks*

**Fig. 2:**
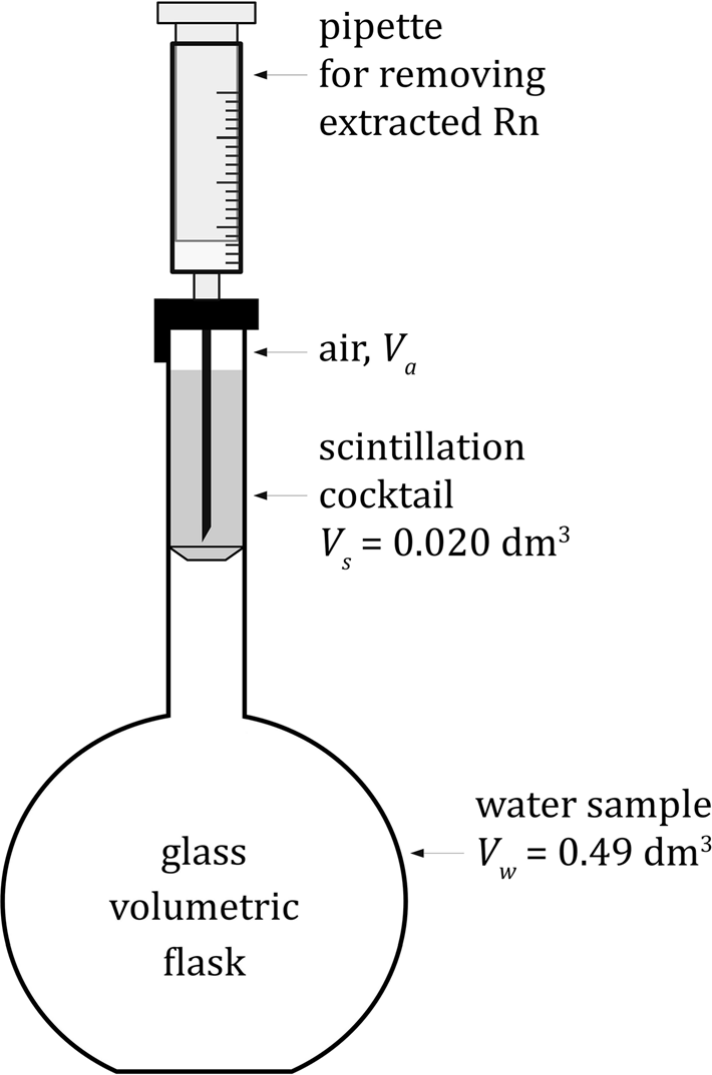
Radon (^222^Rn) extraction system for equilibrium determination of ^226^Ra by LSC method

The Ultima Gold F (UGF) scintillation cocktail was purchased from PerkinElmer Co. (USA). The water samples were collected by a procedure described elsewhere (Bem et al. 2014). The water samples were carefully transferred (to avoid any turbulence) to the 500 cm^3^ glass volumetric flasks. In order to simplify the extraction procedure, 10 cm^3^ of water was removed from the flask, followed by the addition of 20 cm^3^ UGF cocktail. Therefore, the whole volume of the organic phase was placed in the necks of the flasks, and only a small volume of the flask, ca. 5 cm^3^, was filled with air. The tightly capped flasks were vigorously shaken for 5 min and left for half an hour to allow complete phase separation. The schematic representation of the radon extraction system is presented in Fig. 2. From the organic phase, exactly 18 cm^3^ of scintillation cocktail (from the total volume of 20 cm^3^) with the extracted ^222^Rn nuclide was transferred to a typical 20 cm^3^ glass scintillation vial and counted after at least 3 hours delay in a Triathler liquid scintillation counter by a method described elsewhere (Grabowski et al. 2010).

### *Radium (*^*226*^*Ra) in water measurements by liquid scintillation counting*

**Fig. 3:**
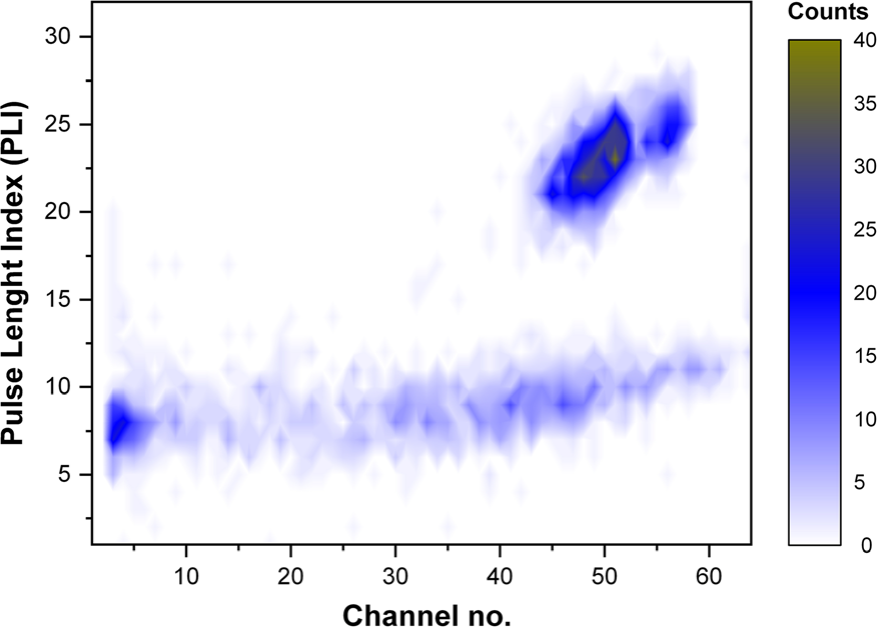
A 2D LSC spectrum collected from ^222^Rn and its progenies in UGF scintillation cocktail in α/β separation mode

The weighted out amounts of water samples from two 1.5 dm^3^ plastic flasks were poured out into a 5 dm^3^ glass beaker and evaporated at 95 °C to a final volume of slightly above 0.5 dm^3^, and if necessary after cooling, 0.1 cm^3^ of concentrated nitric acid was added to dissolve a small amount of the precipitate. The partially evaporated solution was weighed again to calculate the concentration coefficient (*K*_c_ ~ 6). Before pouring out the 490 cm^3^ of preconcentrated water to the glass flask, the electrical conductivity of this solution was measured. After one-month storage time of the samples with the scintillation solution, the radioactive equilibrium between ^222^Rn and ^226^Ra was settled. The whole extraction procedure was the same as for ^222^Rn. The final radioactivity measurements were performed using a Triathler portable liquid scintillation counter in a counting mode with the α/β pulse separation option. This device allows for an efficient separation of the α-pulses coming from the decay of ^222^Rn, ^218^Po and ^214^Po with the total efficiency of ca. 2.4, while background (*B*) in the α region was very low, at a level of 1.7 × 10^–3^ cps (counts per second). The proven efficiency of α/β pulse separation for 18 cm^3^ of the UGF cocktail is shown in Fig. 3.

### *Calculation of the radium (*^*226*^*Ra) activity concentrations in water from LSC*

After radon extraction in the system shown in Fig. 2, the initial ^222^Rn activity from the water phase *A*_o_ will be distributed between three phases, which can be expressed by Eq. (1):

1 $$A_{{\text{o}}} = A_{{\text{w}}} + A_{{\text{s}}} + A_{{\text{a}}}$$

where the lower indexes *w*, *s* and *a* correspond to the radon activities in water, scintillation cocktail and air phases, in Bq. Assuming full equilibrium between the concentrations of ^222^Rn in all phases, according to Nernst’s partition law, one can get two Eqs. (2a) and (2b):

2a $$K_{{{\text{N}}1}} = \frac{{A_{{\text{s}}} /V_{{\text{s}}} }}{{A_{{\text{w}}} /V_{{\text{w}}} }}$$

and

2b $$K_{{{\text{N}}2}} = \frac{{A_{{\text{s}}} /V_{{\text{s}}} }}{{A_{{\text{a}}} /V_{{\text{a}}} }}$$

where *K*_N1_ and *K*_N2_ denote Nernst’s partition coefficients for radon between the organic scintillator and water or the organic scintillator and air phases, respectively, and *V*_w_, *V*_s_ and *V*_a_ are volumes of water, organic scintillator and air, respectively. After combining Eq. (1) with (2a) and (2b), the proper formula for calculating the initial radon activity *A*_o_, in Bq, in the measured water sample can be expressed by Eq. (3):

3 $$A_{{\text{o}}} = A_{{\text{s}}} \left[ {1 + \frac{{V_{{\text{w}}} }}{{V_{{\text{s}}} K_{{{\text{N}}1}} }} + \frac{{V_{{\text{a}}} }}{{V_{{\text{s}}} K_{{{\text{N}}2}} }}} \right].$$

Such calculated activity corresponds to the volume of water equal to *V*_w_, in dm^3^. In order to calculate the absolute radon concentration activity in water *A*_Rn_, in Bq dm^−3^, the value of *A*_o_ should be divided by the volume of water *V*_w_, and *A*_s_ should be replaced by the radon counting rate *I*_s_, radon detection efficiency *ε*_Rn_ as well as the water concentration coefficient *K*_c_, and one can get a final working formula, given by Eq. (4):

4 $$A_{{{\text{Rn}}}} = I_{s} \left[ {1 + \frac{{V_{{\text{w}}} }}{{V_{{\text{s}}} K_{{{\text{N}}1}} }} + \frac{{V_{{\text{a}}} }}{{V_{{\text{s}}} K_{{{\text{N}}2}} }}} \right] \cdot \frac{1}{{V_{{\text{w}}} \varepsilon_{{{\text{Rn}}}} K_{{\text{c}}} }}.$$

After introducing numerical values for *V*_w_ = 0.49 dm^3^, *V*_s_ = 0.02 dm^3^, *V*_a_ = 0.005 dm^3^, *K*_N1_ = 32.4, *K*_N2_ = 8.1, *ε*_Rn_ = 2.4 and *K*_c_ = 6, and taking into account that, experimentally, we can safely take for counting only 18 cm^3^ of pure scintillator phase from its total volume of 20 cm^3^, one can obtain a simple Eq. (5):

5 $$A_{{{\text{Rn}}}} = 0.281I_{{\text{s}}}.$$

The utility of this equation was checked using this method by measuring the set of ^226^Ra standards in water solutions of exactly known activity, *A*_Rn(st)_. We have obtained the experimental calibration coefficient *K*, in Bq dm^−3^ cps^−1^, presented in Eq. (6).

6 $$K = \frac{{A_{{{\text{Ra}}\left( {{\text{st}}} \right)}} }}{{I_{{{\text{st}}}} }} = 0.266 ~ {\text{Bq}} \cdot {\text{dm}}^{ - 3} \cdot {\text{cps}}^{ - 1}.$$

The close values: 0.281 Bq dm^−3^ cps^−1^, that was calculated from Eq. (4) and the experimentally determined calibration coefficient *K* = 0.266 Bq dm^−3^ cps^−1^, confirm the utility of the elaborated method.

### *Validation of the method*

The method was checked in practice with satisfactory results during two Polish interlaboratory tests. One was organized by the Laboratory of Radiometric Expertise of the Institute of Nuclear Physics in Krakow for ^222^Rn in water and the other by the Institute of Nuclear Chemistry and Technology in Warsaw for low ^226^Ra concentrations (below 1 Bq dm^−3^).

A detection limit for ^226^Ra, *L*_DRa_, in Bq dm^−3^, was calculated according to the modified Currie formula (7) (Currie 1968) taking into account Eqs. (4) and (6):

7 $$L_{{{\text{DRa}}}} = 0.266\left[ {\frac{2.71 + 3.29\sqrt B }{t}} \right]$$

where *B* denotes the blank in counts (background counts), *t* is the time of counting, in seconds, and 0.266 is a calibration coefficient for this method, in Bq dm^−3^ cps^−1^. For the standard time of counting of 3600 s and background *B* = 6 cts, the calculated detection limit for this method was found to be *L*_DRa_ = 0.001 Bq dm^−3^. The determination limit, *L*_qRa_*,* with a relative error not exceeding 10% was calculated according to Eq. (8).

8 $$L_{{{\text{qRa}}}} = \frac{0.266 \cdot 50}{t}\left[ {1 + \left( {1 + \frac{B}{25}} \right)^{1/2} } \right].$$

After substituting the same values of *B* and *t*, the calculated determination limit was found to be *L*_qRa_ = 8 × 10^–3^ Bq dm^−3^.

### *Correction for the activity of extracted *^*222*^*Rn from water with enhanced salinity after the preconcentration step*

**Fig. 4:**
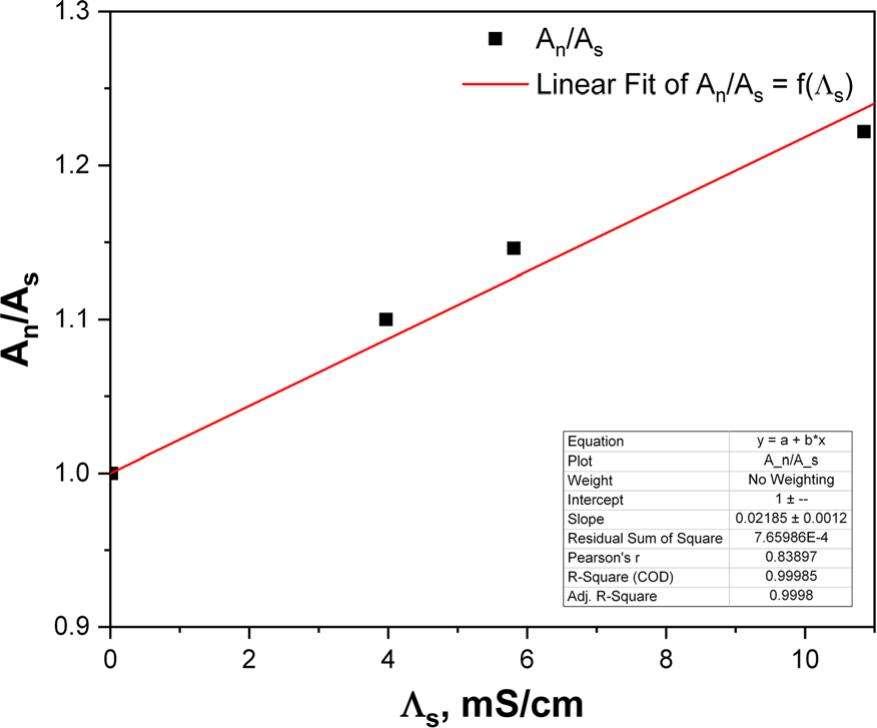
Relative extraction efficiency of ^222^Rn isotope from water solutions of different salinity

The dependence of the water/air partition coefficient of radon, *K*_w/air_, on the salinity of the water phase has been documented for a broad range of salt concentrations (Schubert et al. 2012). Although related changes in radon solubility in typical situations, including seawater samples, do not exceed 10%, one should check how the water salinity influences the extraction of radon from the water to the UGF phase. In practice, the water salinity can be well characterized by its electrical conductivity. After preparing a set of the standard ^222^Rn solutions in water with dissolved MgCl_2_ in the range from 0 to 10 g dm^−3^, conductivity of these solutions was measured, and finally, the ^222^Rn nuclide was extracted to the UGF cocktail. The dependence of the ratio of the ^222^Rn activity extracted from the distilled water (*A*_n_) to these extracted from saline solutions (*A*_s_) vs. the water conductivity is shown in Fig. 4. The working expression, that describes the dependence of water conductivity on the normalized value of activity *A*_n_, which corresponds to a pure water takes the form expressed by Eq. (9):

9 $$A_{{\text{n}}} = A_{{\text{s}}} \left( {1 + 0.022\Lambda_{{\text{s}}} } \right).$$

*Λ*_s_ is the conductivity of the water sample, in mS cm^−1^.

### *Determination of radon in soil gas in the vicinity of underground water supply units*

The ^222^Rn nuclide concentration in soil gas was determined at four points around each water supply stations by the method described elsewhere (Bem et al. 2017, 2020). The soil was sampled on days when there was no rain, usually at noon, using a typical hollow tube probe from 100 cm below ground level by 10 min of sucking about 3 dm^3^ of gas, and passing it directly through 16 cm^3^ of water-immiscible liquid scintillator in standard 20 cm^3^ scintillation vials. The activity of the absorbed radon nuclides was measured using the Triathler device. The radon in soil gas concentration, *C*_Rn_, in Bq m^−3^, can be calculated from an empirical formula given by Eq. (10) (Bem et al. 2017):

10 $$C_{{{\text{Rn}}}} = K_{{\text{e}}} I_{{{\text{SC}}}} e^{0.693\Delta t/91.8}$$

where *K*_e_ is the calibration coefficient of the method, in Bq dm^−3^ cps^−1^, *I*_SC_ is a measured radon counting rate, in cps, and *Δt* is the delay time between the end of radon in soil absorption and the end of activity measurements, in hours. The *K*_e_ calibration coefficient can be calculated from Eq. (11) taking into account temperature, *T*, of the radon absorption in scintillation cocktail (Bem et al. 2017).

11 $${\text{ln}}\,K_{{\text{e}}} = 12.03 - \frac{1180}{T}.$$

## Results and discussion

### ^*222*^*Rn activity concentrations in water and soil gas around water treatment plants (WTP)*

The basic parameters of water in particular supply sites, the ^222^Rn activity concentration in water before and after its treatment, as well as the average concentration in soil gas around these plants are shown in Table 1.

**Table 1:** Radon (^222^Rn) and radium (^226^Ra) activity concentration in measured samples from Kalisz district of Poland; ^*^relative errors for determination of soil gas radon concentration below 20%, n.a. – data not available

No	Site	Water parameters			^222^Rn conc., Bq dm^−3^		^222^Rn removal, %	^226^Ra activity in water, Bq dm^−3^	(^226^Ra/^222^Rn) × 10^3^ (before WTP)	^222^Rn in soil gas^*^, Bq dm^−3^
		pH	Conductivity, μS cm^−1^	Hardness, mg CaCO_3_/dm^3^	Before WTP	After WTP				
1	Dzierzbin	7.4	288	146	2.53 ± 0.06	1.29 ± 0.10	49	(3.2 ± 0.6) × 10^–3^	1.26	2.48
2	Korzeniew	7.4	240	120	1.64 ± 0.13	1.46 ± 0.03	11	(4.6 ± 0.7) × 10^–3^	2.80	2.25
3	Kościelec	7.2	628	266	7.93 ± 0.04	4.07 ± 0.03	49	(24.8 ± 1.4) × 10^–3^	3.13	33.3
4	Dębe	7.6	441	226	1.98 ± 0.01	1.17 ± 0.01	41	(7.1 ± 0.8) × 10^–3^	3.59	7.82
5	Janków Goliszewski	7.5	650	326	2.03 ± 0.04	1.46 ± 0.13	28	(5.5 ± 0.7) × 10^–3^	2.71	5.73
6	Kokanin	7.3	601	384	4.65 ± 0.06	3.95 ± 0.04	15	(12.4 ± 1.0) × 10^–3^	2.67	35.8
7	Pólko	7.4	459	234	3.21 ± 0.13	2.06 ± 0.04	36	(7.9 ± 0.8) × 10^–3^	2.46	18.5
8	Pawłówek	7.4	790	360	9.01 ± 0.03	4.97 ± 0.11	45	(26.2 ± 1.5) × 10^–3^	2.80	34.4
9	Tłokinia Wielka	7.6	752	360	4.07 ± 0.06	2.56 ± 0.04	37	(11.4 ± 1.0) × 10^–3^	2.80	9.52
10	Opatówek	7.7	502	247	4.44 ± 0.04	3.41 ± 0.06	23	(12.5 ± 1.0) × 10^–3^	2.82	8.06
11	Brzeziny	7.2	279	122	4.67 ± 0.11	3.15 ± 0.18	33	(11.2 ± 1.0) × 10^–3^	2.40	6.01
12	Wolica	7.4	432	208	4.23 ± 0.03	2.62 ± 0.06	38	(12.1 ± 1.0) × 10^–3^	2.86	7.83
13	Czempisz	7.8	245	124	7.28 ± 0.04	5.17 ± 0.10	29	(19.9 ± 1.3) × 10^–3^	2.73	29.8
14	Pietrzyków	7.3	582	248	3.62 ± 0.01	2.74 ± 0.00	24	≤ LLD	≤ 0.97	13.1
15	Michałów II	8.0	438	190	2.67 ± 0.04	1.65 ± 0.06	38	(4.4 ± 0.7) × 10^–3^	1.65	0.80
16	Kamień	7.2	598	293	3.75 ± 0.07	3.58 ± 0.01	5	(5.1 ± 0.7) × 10^–3^	1.36	2.02
17	Żelazków	7.8	666	308	1.82 ± 0.11	1.54 ± 0.01	15	(5.4 ± 0.7) × 10^–3^	2.97	21.8
18	Michałów	7.5	984	408	1.52 ± 0.14	0.81 ± 0.13	47	(3.5 ± 0.6) × 10^–3^	2.30	9.40
19	Rychnów	7.7	657	306	3.83 ± 0.04	2.30 ± 0.01	40	(20.3 ± 1.3) × 10^–3^	5.30	5.64
20	Blizanów	7.7	679	268	3.38 ± 0.08	2.17 ± 0.00	36	(14.7 ± 1.1) × 10^–3^	4.35	2.12
21	Strzałków	7.3	482	234	3.20 ± 0.07	1.65 ± 0.14	48	(10.1 ± 0.9) × 10^–3^	3.16	23.7
22	Stawiszyn	7.4	502	231	4.02 ± 0.13	3.73 ± 0.08	7	(18.2 ± 1.2) × 10^–3^	4.53	5.63
23	Pieczyska	8.1	248	122	3.45 ± 0.07	1.46 ± 0.13	58	(12.4 ± 1.0) × 10^–3^	3.59	9.89
24	Piątek Wielki	7.5	447	234	3.15 ± 0.04	2.34 ± 0.03	26	(9.1 ± 0.9) × 10^–3^	2.89	4.04
25	Dębsko	7.2	497	319	6.41 ± 0.17	4.10 ± 0.01	36	(11.2 ± 1.0) × 10^–3^	1.75	32.5
26	Koźminek	7.3	592	242	4.53 ± 0.01	3.76 ± 0.03	17	(8.6 ± 0.9) × 10^–3^	1.90	19.7
27	Lisków	7.6	409	182	4.52 ± 0.04	3.28 ± 0.08	27	(11.4 ± 1.0) × 10^–3^	2.52	14.3
28	Morawin	7.3	444	198	6.91 ± 0.03	5.02 ± 0.06	27	(12.4 ± 1.0) × 10^–3^	1.79	32.7
29	Cienia II	7.6	377	182	3.53 ± 0.04	3.10 ± 0.13	12	(10.8 ± 1.0) × 10^–3^	3.06	12.4
30	Radliczyce	7.4	513	294	6.77 ± 0.04	4.46 ± 0.06	34	(21.7 ± 1.3) × 10^–3^	3.21	29.0
31	Jastrzębniki	7.6	817	360	6.67 ± 0.04	4.14 ± 0.17	38	(13.5 ± 1.1) × 10^–3^	2.02	31.4
32	Lipe	7.6	659	299	3.90 ± 0.07	3.84 ± 0.06	2	(12.6 ± 1.0) × 10^–3^	3.23	5.49
33	Staw	7.6	518	252	4.41 ± 0.03	3.08 ± 0.06	30	(15.1 ± 1.1) × 10^–3^	3.42	14.0
34	Zbiersk	7.6	350	168	2.09 ± 0.04	1.09 ± 0.06	48	(9.6 ± 0.9) × 10^–3^	4.59	6.07
35	Zbiersk Kolonia	7.7	360	130	1.60 ± 0.04	0.66 ± 0.04	59	(20.3 ± 1.3) × 10^–3^	12.69	5.39
36	Zagorzyn	7.5	761	368	1.68 ± 0.10	1.66 ± 0.01	1	(20.3 ± 1.3) × 10^–3^	12.08	10.6
37	Jastrzębniki II	n.a	n.a	n.a	3.16 ± 0.00	2.19 ± 0.10	31	(11.1 ± 1.0) × 10^–3^	3.51	9.65
38	Biała	7.4	405	196	3.25 ± 0.03	2.18 ± 0.04	33	(14.9 ± 1.1) × 10^–3^	4.58	11.6
39	Moskurnia	7.4	624	222	6.02 ± 0.07	3.27 ± 0.03	46	(11.1 1.0) × 10^–3^	1.84	14.5
40	Rajsko	7.5	675	372	0.28 ± 0.14	0.28 ± 0.13	0	(3.6 ± 0.6) × 10^–3^	12.86	8.98
41	Mroczki Wielkie	7.2	500	226	2.44 ± 0.06	1.30 ± 0.04	47	(12.5 ± 1.0) × 10^–3^	5.12	16.9
42	Danowiec	7.5	239	118	0.99 ± 0.13	0.92 ± 0.04	7	≤ LLD	≤ 3.54	0.82
43	Iwanowice	7.6	332	166	3.95 ± 0.14	3.56 ± 0.10	10	(11.4 ± 1.0) × 10^–3^	2.89	16.3
44	Szczytniki	7.5	386	196	8.31 ± 0.08	4.19 ± 0.10	50	(17.9 ± 1.2) × 10^–3^	2.15	22.6
45	Kalisz Lis	7.4	541	266	5.26 ± 0.06	1.26 ± 0.06	76	(22.4 ± 1.3) × 10^–3^	4.26	4.80
46	Kalisz Fabryczna	7.3	658	265	0.99 ± 0.14	0.50 ± 0.11	49	(24.1 ± 1.4) × 10^–3^	24.34	4.89
47	Kalisz Poznańska	7.2	917	387	6.48 ± 0.01	2.48 ± 0.04	62	(22.9 ± 1.4) × 10^–3^	3.53	9.85
48	Kalisz Warszawska	7.4	683	283	5.47 ± 0.01	4.36 ± 0.06	20	(21.9 ± 1.3) × 10^–3^	4.00	30.1
49	Ceków	n.a	n.a	n.a	4.15 ± 0.04	2.46 ± 0.00	41	(12.3 ± 1.0) × 10^–3^	2.96	16.5
50	Piegonisko Wieś	n.a	n.a	n.a	5.53 ± 0.13	1.91 ± 0.01	65	(28.5 ± 1.5) × 10^–3^	5.15	26.5

**Fig. 5:**
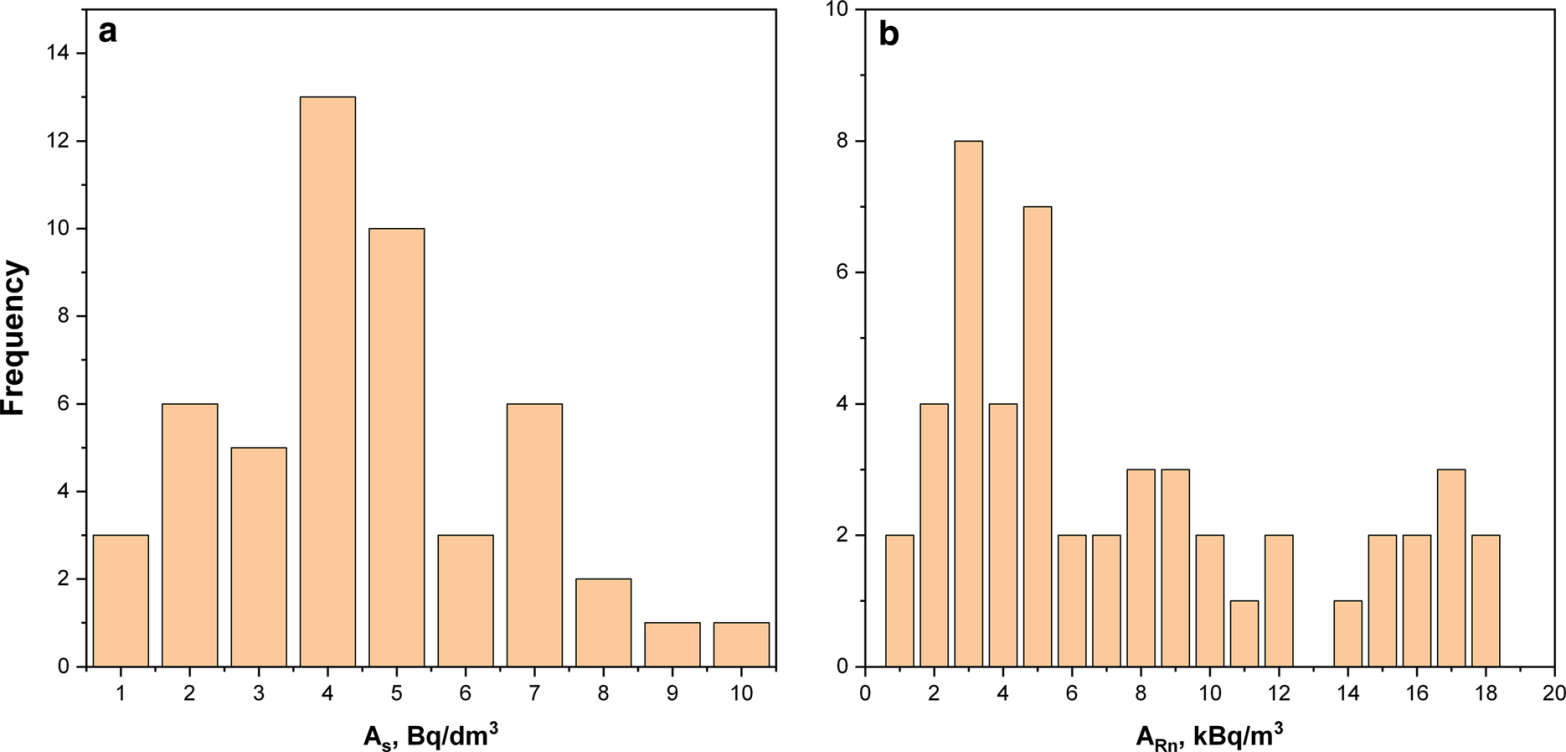
Distribution of ^222^Rn activity concentrations in crude water samples (**a**) and in adjacent soil gas (**b**) from 50 WTPs in Kalisz region

In the majority of the WTPs in the examined region, after aeration, water passes through mineral filters for removal of suspended matter, and at least one-third of the radon nuclide is eliminated after treatment. The radon activity distribution in crude water samples is more uniform than for radon in soil gas (cf. Fig. 5). The average radon activity concentration in the water coming in was 4.03 Bq dm^−3^, whereas the average concentration of this radionuclide in the water supplied for drinking and domestic use was only 2.61 Bq dm^−3^. The latest value is close to the previously determined average radon activity concentration in this area and very close to those obtained for drinking water samples from other parts of Poland (Chruścielewski and Kamiński 1999; Karpińska et al. 2010; Kochowska et al. 2004), except for Southern Poland, in the Sudety mountain areas, where increased radon activity in underground water of 444.9 Bq dm^−3^ was observed (Kusyk and Mamont-Ciesla 2002; Przylibski et al. 2014).

As it is evident from Table 1, the measured conductivities for the examined drinking water samples ranged from 0.25 to 0.95 mS cm^−1^. Therefore, according to Eq. (9) the corrections for the ^222^Rn activity determinations in drinking water samples were negligible (< 2.1%). The conductivities of sixfold preconcentrated water samples were two to three times higher of those before evaporation, but they did not exceed the value of 3 mS cm^−1^ and the corresponding corrections for calculation of the ^226^Ra activity concentration were below 8%.

**Fig. 6:**
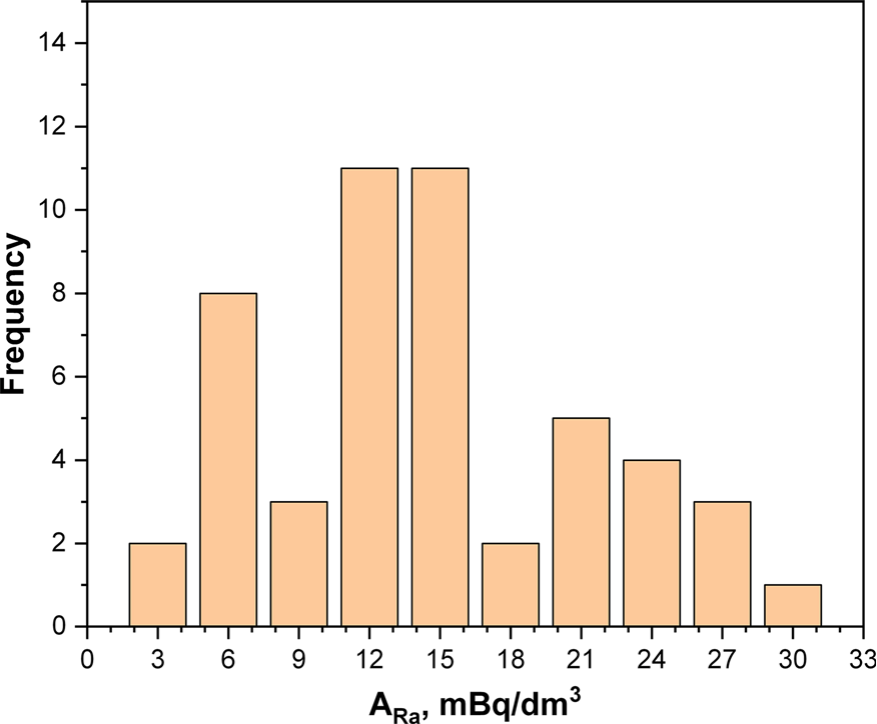
Distribution of ^226^Ra activity concentrations for water samples from 50 WTPs in Kalisz region

The distribution of ^226^Ra activity concentrations for water samples from all 50 WTPs in Kalisz is shown in Fig. 6. As expected, the measured values for this radionuclide were low, within the range from 10^–3^ Bq dm^−3^ (close to the detection limit) to the maximum value of 28.5 × 10^–3^ Bq dm^−3^. The geometric mean and median values were close: 10.3 and 12.2 × 10^–3^ Bq dm^−3^, respectively (cf. Table 2). These values are much lower than those reported for the Sudety (median 0.08 Bq dm^−3^) or Carpathian region (median 0.62 Bq dm^−3^) (Kusyk and Mamont-Ciesla 2002), and they are comparable with the Roztocze area in southern Poland, where the maximum values of ^226^Ra activity concentrations in underground water reached a level of 0.07 Bq dm^−3^. By contrast, for the majority of samples in Roztocze region, the activity concentrations were below the detection limit of the method used, i.e., < 0.06 Bq dm^-3^.

**Table 2:** Parameters of ^222^Rn and ^226^Ra activity distributions in the measured water samples

Parameter	^222^Rn activity in water, Bq dm^−3^		^226^Ra activity in water, Bq dm^−3^	^222^Rn activity in soil gas, Bq dm^−3^
	Before WTP	After WTP		
Minimal activity	0.28	0.28	3.20 × 10^–3^	0.80
Maximal activity	9.01	5.17	28.5 × 10^–3^	35.8
Arithmetic mean	4.03	2.61	13.0 × 10^–3^	14.1
Standard dev. of mean	2.02	1.30	6.67 × 10^–3^	10.5
Geometric mean	3.44	2.22	11.1 × 10^–3^	10.0
Median	3.87	2.47	12.2 × 10^–3^	10.3

**Fig. 7:**
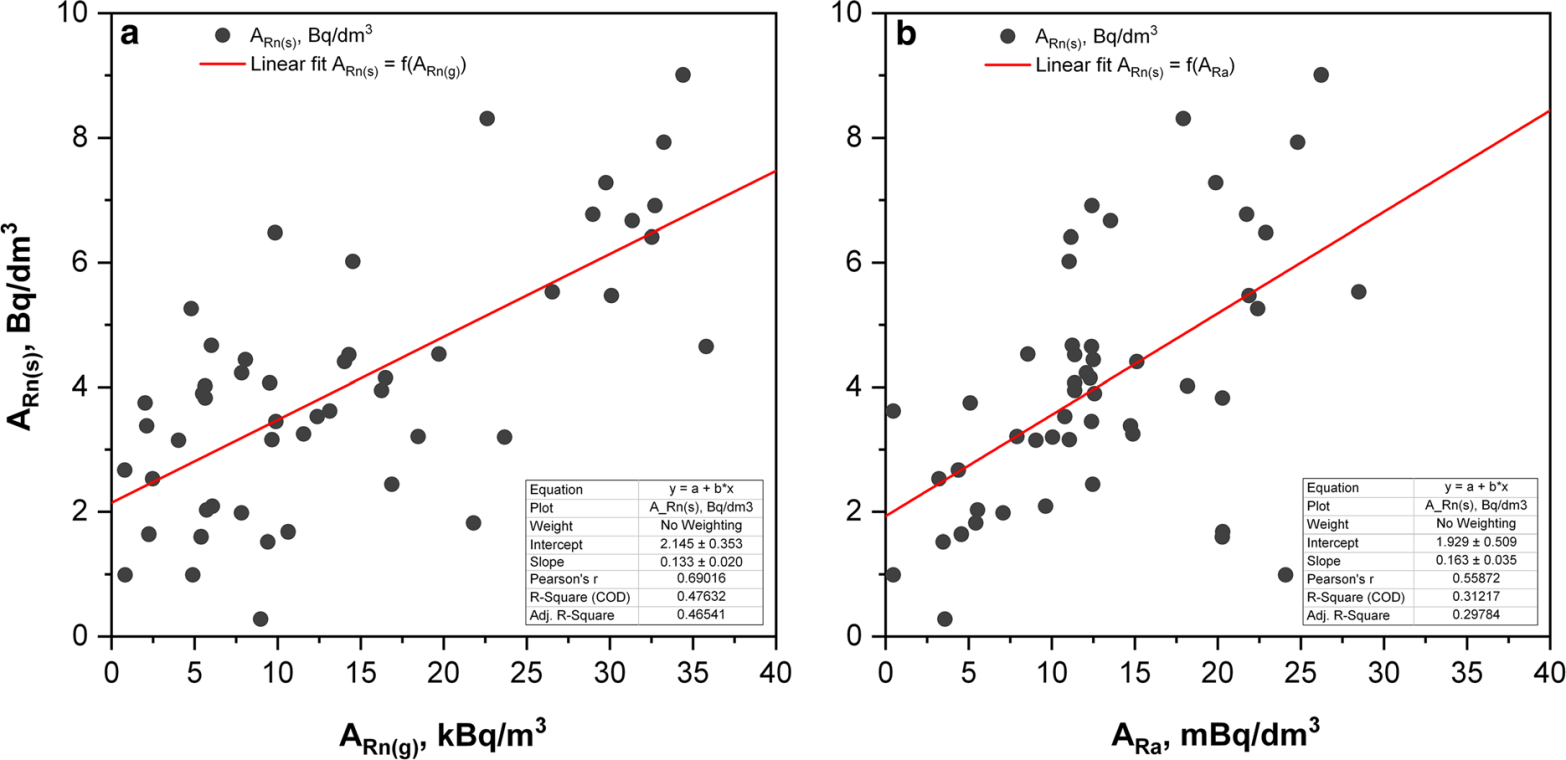
Relationships between ^222^Rn activity in water and in surface soil gas (**a**) and ^226^Ra nuclide in water (**b**)

Interestingly, a moderate positive relationship (*R*^*2*^ = 0.48) between radon in water and radon in surface soil gas concentrations is observed in Fig. 7a, although underground water is pumped from reservoirs situated about 100 m (average) below the land surface. It can be explained by the fact, that the majority of the underground water sources in this area were drilled mostly in Upper and Middle Jurassic, as well as Upper Albian–Turean or Coniacian and Santonian geological formations, with low concentrations of uranium and radium, and consequently with low radon influx into existing water reservoirs. It was also confirmed by the relatively low radon in soil gas concentration, which has a geometric mean value of 10 kBq m^−3^. The primary source of Ra and Rn in groundwater has recoiled from parent radionuclides on fracture surfaces, which is balanced mostly by the behavior of ^226^Ra^2+^ ions in particular hydrochemical conditions, bedrock geology and its adsorption on the surface of minerals surrounding water reservoir. On the other side, radon concentrations were not correlated with most hydrochemical water components, including uranium concentration. The radon emanation from the soil is controlled not only by lithology and associated radium content of the rocks and soil, but also by structural zones which help in the easy migration of radon from the deeper parts of the soil to surface earth's crust. Therefore, a wide range of the ^226^Ra/^222^Rn ratios from 2.26 × 10^–6^ to 9.70 with a median value of 0.0035 for groundwater and springs has been reported (Girault et al. 2018). However, the radon gas together with other gases in the soil can, under favorable circumstances, migrate faster to the earth’s surface. Therefore, its concentration in surface soil at the 1 m depth does not respond exactly to its concentration in the higher depths of hundred meters around the water reservoirs. This is a reason for the observed a very weak correlation. Moreover, the existence of the positive free coefficient in the linear correlation equation between radon in water and in soil gas levels indicates on a possible higher radon in soil concentrations in the deeper soil layers. However, a weaker correlation (*R*^*2*^ = 0.31) between the activity concentration of ^226^Ra and ^222^Rn was observed (cf. Fig. 7b). It can be explained by the observation that Ra’s adsorption onto aquifer surfaces has a dominant control on its behavior in groundwater. Therefore, radium concentrations may not correlate with either ^222^Rn (a measure of recoil supply) or total dissolved solids since the controlling factor was the physicochemical properties of the solid phase around the water reservoirs (Almeida et al. 2004; Porcelli and Swarzenski 2003). Thus, it is interesting to check other possible correlations, which could be taken into account for the physicochemical parameters of the water sample. The results of such a multivariate correlation analysis are shown in Table 3. As expected, the results confirmed (*P-value* < 0.05) that one can expect such weak or moderate strength correlation only between previously described activity concentrations of ^222^Rn in soil and raw water, or ^226^Ra and ^222^Rn in water. In all other circumstances, the *P-values* significantly exceeded 0.05, which suggests that other independent factors coexisting with soil gas radon and ^226^Ra concentration in water are unimportant. Table 3 presents also other parameters obtained as an output from multivariate analysis: *R* correlation coefficient (*Multiple R*) and *R-square* (*R*^*2*^) value, degrees of freedom (*df*), sum of squares (*SS*), mean squared errors (*MS*) and *F-statistics* (*F*) values (F test used to test the hypothesis that the slope of the independent variable is zero). The calculated values of the *t-Statistic* coefficients were found to be significantly higher, in comparison with *t-Statistic* values for other variables, only for ^226^Ra concentration in water and ^222^Rn in soil gas, which confirms the conclusions from the analysis of *P-value* parameters.

**Table 3:** Results of multivariate correlation analysis for ^222^Rn concentration in crude (not pretreated) water; df—degrees of freedom, SS—sum of squares, MS—mean squared errors, F—value of F-statistic

Regression statistics						
Multiple *R*	0.875					
*R* square (*R*^2^)	0.765					
Standard error	1.219					
Count of X variables	14					
Observations	43					
Adjusted *R *square	0.647					

Analysis of variance (ANOVA)	*df*	SS	MS	*F*	Significance *F*	
Regression	14	135.38	9.67	6.50	1.39 × 10^–5^	
Residual	28	41.62	1.49			
Total	42	177.00				

Parameters	Coefficients	Std. error	*t*-Statistic	*P-*value	Lower 95%	Upper 95%
Intercept	10.436	9.511	1.097	0.282	− 9.045	29.918
^226^Ra conc. in water, Bq/dm^3^	421.7	140.8	2.995	5.68 × 10^–3^	133.3	710.1
^222^Rn in soil gas, Bq/dm^3^	0.115	0.024	4.751	5.49 × 10^–5^	0.065	0.164
pH	− 1.202	1.217	− 0.988	0.332	− 3.695	1.290
Conductivity, μS/cm	0.005	0.006	0.872	0.391	− 0.007	0.017
Hardness, mg CaCO_3_/L	− 0.017	0.010	− 1.673	0.105	− 0.038	0.004
Chlorides, mg/L	0.036	0.030	1.215	0.235	− 0.025	0.098
Manganese, μg/L	− 0.010	0.018	− 0.563	0.578	− 0.047	0.027
Sulfates, mg/lL	− 0.010	0.013	− 0.769	0.448	− 0.038	0.017
Fluorides, mg/L	1.330	1.112	1.195	0.242	− 0.949	3.608
Sodium, mg/L	0.006	0.024	0.256	0.799	− 0.043	0.056
Nickel, μg/L	0.056	0.051	1.091	0.284	− 0.049	0.161
Copper, mg/L	− 2.484	1.251	-1.987	0.057	− 5.046	0.077
Cadmium, μg/L	− 0.743	0.487	-1.527	0.138	− 1.740	0.254
Lead, μg/L	0.346	0.356	0.970	0.340	− 0.384	1.075

### *Simultaneous determination of *^*226*^*Ra and *^*228*^*Ra in water samples by γ-spectrometry*

According to the EURATOM Directive (European Commission 2013), monitoring water intended for human consumption for the indicative dose (*ID*) should be carried out where a source of artificial or elevated natural radioactivity is present and cannot be shown, based on other representative monitoring programs or other investigations, that the level of *ID* is below the parametric value of 0.1 mSv year^−1^. Member States may use screening strategies for gross alpha and gross beta activity (European Commission 2013) to monitor for the parametric indicator value for the *ID*. An alternative approach to evaluate the *ID* is to compare the activity concentration of the main radionuclides present in water with its proposed derived concentrations for radioactivity in water, whose intake corresponds to the annual effective dose of 0.1 mSv. Among natural radionuclides present in underground water, ^40^K and uranium isotopes can be easily determined by chemical methods, and their concentrations in these water samples lead to internal doses in the range below 1 µSv (Dinh Chau et al. 2011; UNSCEAR 2000). The value of the proposed derived concentration for ^228^Ra is lower (0.2 Bq dm^−3^) than that for ^226^Ra (0.5 Bq dm^−3^). The ^228^Ra nuclide decays by emitting beta particles, and it can be determined by liquid scintillation technique after settling its radioactive equilibrium (at least two days) with parent nuclide ^228^Ac. However, this method is time-consuming and needs use of very expensive liquid scintillation counters with ultra-low background in beta counting channel. Fortunately, radionuclide ^228^Ac emits β-particles with accompanying γ-radiation. Therefore, γ-spectrometry is also often used for its determination.

However, in this method, a preconcentration step by coprecipitating radium with a mixture of barium and lead sulfates from at least 5 dm^3^ of water is necessary. In order to evaluate the efficiency of radium precipitation, a marker of ^133^Ba radionuclide was added. After overnight storage, the solution with precipitated barium and lead sulfates was filtered, dried and finally transferred with a filter to a small plastic container and kept closed for one month before γ-spectrometry analysis for simultaneous determination of ^133^Ba, ^226^Ra (from γ-lines of ^214^Pb and ^214^Bi) and ^228^Ra (from ^228^Ac lines). Details of spectrometry analysis have been described elsewhere (Bem et al. 2004). However, a drawback of this method is that it is more time-consuming and possesses higher detection limits in comparison with LSC: 0.01 Bq dm^−3^ for ^226^Ra and 0.015 Bq dm^−3^ for ^228^Ra. In case of water samples analyzed in this study, the results obtained with satisfactory accuracy were observed only for nine water sampling sites. A comparison of these results with ^226^Ra activity concentration measured by LSC methods is shown in Table 4.

**Table 4:** Comparison of ^226^Ra and ^228^Ra determinations obtained due to LSC and γ-spectrometry techniques

No	Site	Activity concentration of radium by, Bq dm^−3^:			^228^Ra/^226^Ra activity ratio
		LSC (^226^Ra)	γ-spectrometry (^226^Ra)	γ-spectrometry (^228^Ra)	
2	Korzeniew	4.6 × 10^–3^	≤ 10 × 10^–3^	18.6 × 10^–3^	4
9	Tłokinia Wielka	11.4 × 10^–3^	11.0 × 10^–3^	≤ 15 × 10^–3^	~ 1
10	Opatówek	12.5 × 10^–3^	19.5 × 10^–3^	18.1 × 10^–3^	1.4
26	Koźminek	8.6 × 10^–3^	13.4 × 10^–3^	≤ 15 × 10^–3^	~ 1
29	Cienia II	10.8 × 10^–3^	≤ 10 × 10^–3^	24.3 × 10^–3^	2.3
33	Staw	15.1 × 10^–3^	10.0 × 10^–3^	24.3 × 10^–3^	1.6
36	Zagorzyn	20.3 × 10^–3^	29.0 × 10^–3^	≤ 15 × 10^–3^	< 1
41	Mroczki Wielkie	12.5 × 10^–3^	≤ 10 × 10^–3^	23.4 × 10^–3^	1.9
44	Szczytniki	17.9 × 10^–3^	20.5 × 10^–3^	≤ 15 × 10^–3^	< 1

In the case of water samples analyzed in this study, the results obtained with satisfactory accuracy were observed for seven water sampling sites for ^226^Ra and five sites for ^228^Ra, only. Generally, radium concentrations, determined by γ-spectrometry for both radionuclides, are very low, below 0.03 Bq dm^−3^, and a satisfactory consistency for both methods was observed for ^226^Ra. The activity ratio of ^228^Ra/^226^Ra in the five examined groundwater samples ranged from ca. 1 to 4 and was similar to that observed for the Sudety region of Poland (between 0.099 and 2.059). Generally, for ^226^Ra, a satisfactory consistency for both methods was observed. Unfortunately, the activity concentrations of ^228^Ra nuclide could be determined for only five sites. However, those scarce data for ^228^Ra are close to the results published for underground water samples for the Sudety region, where the analysis of sampling results for water from 55 intakes (including deep well with high values of total dissolved solids) showed median concentrations of 0.051 Bq dm^−3^ for ^226^Ra and 0.048 Bq dm^−3^ for ^228^Ra (Przylibski et al. 2002). The activity ratio of ^228^Ra/^226^Ra in the examined groundwater samples from the Sudety region ranges between 0.099 and 2.059, which is also close to these radionuclide ratios observed in the Kalisz region. It should be noticed that for the Outer Carpathian region of Poland, the activity concentrations of ^228^Ra nuclide in the majority of the measured underground water samples were below the detection limit of the method used, i.e., 0.03 Bq dm^−3^ (Walencik et al. 2010).

### *Effective dose calculation*

The total committed effective dose, *E*_Rn_, for the general population caused by the occurrence of radon in drinking water and its domestic use is the sum of the effective doses due to radon ingestion with water, *E*_Rn,ing_, and inhalation from waterborne radon, *E*_Rn,inh_. The effective dose from water ingestion was calculated from Eq. (12) (UNSCEAR 2000):

12 $$E_{{\text{Rn,ing}}} = {\text{ DCF}}_{{{\text{ing}}}} A_{{{\text{Rn}}}} V_{{\text{w,ing}}}$$

where *DCF*_ing_ is a dose conversion factor (or dose coefficient), in Sv Bq^−1^, which corresponds to the effective dose due to ingestion of the unit activity of the particular radionuclide, *A*_Rn_ is the average radon activity in a drinking water, in Bq dm^−3^, and *V*_w,ing_ is the estimated annual volume of water consumed directly from the tap, in dm^3^. There is some controversy concerning the numerical values of dose conversion factors for radon as well as for the volume of annual ingested water. Since radon is readily lost from water by heating or boiling, the total annual water intake of 60 dm^3^ for the consumption of tap water was proposed in the UNSCEAR 2000 Report (UNSCEAR 2000), instead of usually used 730 L for total water consumption, and this value of *V*_w,ing_ has been used in this work. For the *DCF*_ing_, recently, a more conservative value of 1 × 10^–8^ Sv Bq^−1^ has also been recommended and generally accepted (Kendall and Smith 2002). Therefore, after substituting these values in Eq. (12), for the average activity concentration of ^222^Rn in water *A*_Rn_ = 2.22 Bq dm^−3^ one can get the effective dose *E*_Rn,ing_ = 1.3 μSv year^−1^.

The dose from inhalation of waterborne radon can be calculated from Eq. (13) (UNSCEAR 2000):

13 $$E_{{\text{Rn,inh}}} = {\text{DCF}}_{{{\text{inh}}}} A_{{{\text{Rn}}}} TFt$$

where *DCF*_Rn,inh_ is the radon dose conversion factor for radon inhalation, *DCF*_Rn,inh_ = 16.8 × 10^–9^ Sv m^−3^ Bq^−1^ h^−1^, *A*_Rn_ is the average radon in water activity concentration in Bq dm^−3^, *T* is the radon transfer from water to air coefficient, *T* = 0.1 dm^3^ m^−3^ (Harley et al. 2014), *t* is the average annual indoor occupancy, in hours (*t* = 7000 h), and *F* is the indoor radon daughters equilibrium factor, *F* = 0.4. The value of 16.8 10^–9^ Sv m^−3^ Bq^−1^ h^−1^ for *DCF*_Rn,inh_ was recently recommended by the ICRP (Paquet et al. 2017). Therefore, for the average radon in water concentration of 2.22 Bq dm^−3^, the effective dose from inhalation of waterborne radon is equal to *E*_Rn,inh_ = 16.8 × 10 ^−9^ × 2.22 × 0.1 × 7000 × 0.4 = 10.4 μSv year^−1^.

The ^226^Ra concentrations in the measured water samples were in the range of 0.004 to 0.0285 Bq dm^−3^. For the average geometric concentration of this radionuclide, i.e., 10.3 × 10^–3^ Bq dm^−3^, the corresponding annual effective dose from its ingestion can also be calculated from Eq. (12), but the *DCF*_Ra,ing_ = 2.8 × 10^–7^ Sv Bq^−1^ for adults and yearly water consumption *V*_w,ing_ should be taken as 730 dm^3^. Such a calculated value for committed effective dose from radium intake with water is *E*_Ra,ing_ = 2.1 μSv year^−1^.

It confirms the suggestion that for the majority of domestic circumstances, the effective doses from inhaling waterborne radon are one order higher than those from radon ^222^Rn or radium ^226^Ra nuclide ingestions with water. However, it should be clearly stated that these three doses are not significant in comparison with that from indoor radon inhalation in the Kalisz area, where the geometric average indoor radon concentration is around 30 Bq m^−3^ and the effective dose for Kalisz inhabitants is *E*_inh_ = 1.28 mSv (corrected by a factor of 3.5 for 7000 h indoor radon exposition in houses), i.e., over 100 times higher (Bem et al. 2013).

## Conclusions

Liquid scintillation counting with separation of α/β pulses makes it possible to achieve a very low detection limit, i.e., *LD* = 0.005 Bq dm^−3^ for ^222^Rn nuclide extracted directly from 0.5 dm^3^ water samples to 20 cm^3^ of water-immiscible scintillation cocktail Ultima Gold F.

Sixfold preconcentrated water samples by evaporation and kept in 0.5 dm^3^ flasks with the same cocktail over one month can be used to determine the ^226^Ra nuclide with a detection limit of < 1 × 10^–3^ Bq dm^−3^. Such a low limit makes it possible to measure ^226^Ra activity concentrations in both underground and surface water samples.

The average activity concentrations of ^222^Rn and ^226^Ra nuclides in the water samples supplied for domestic use, including as a drinking water, after its treatment in the 50 water treatment plants in the Kalisz district were low: 2.22 Bq dm^−3^ and 10.3 × 10^–3^ Bq dm^−3^, respectively. Therefore, the calculated committed effective doses from ingestion or domestic use of water for the population of the Kalisz area are negligible.

A pilot study to determine activity concentrations of ^228^Ra in the same water sample showed that the activity ratios of ^228^Ra/^226^Ra were in the range < 1 to 2.4. It is an important indicator to evaluate the indicative dose proposed in the EU Directive for water intended for human consumption. For example, if the activity of ^226^Ra does not exceed 0.05 Bq dm^−3^, one can assume with high probability that the activity concentration of ^228^Ra will be below the derivative value of 0.2 Bq dm^−3^ for this radionuclide. It makes it possible to avoid the time-consuming and expensive determination of ^228^Ra radionuclide.

Comparing ^222^Rn in crude water samples with ^226^Ra activity concentration shows a weak correlation between these two parameters (*R*^*2*^ = 0.31). On the other hand, a medium strength correlation (*R*^*2*^ = 0.48) has been observed between ^222^Rn concentrations in underground water and adjacent soil gas. Therefore, radon in soil measurements can give valuable information not only about prospective indoor radon concentration in houses in this area but also expected levels of ^222^Rn in underground water.
